# Serum Branched-Chain Amino Acids and Long-Term Complications of Liver Cirrhosis: Evidence from a Population-Based Prospective Study

**DOI:** 10.3390/nu16142295

**Published:** 2024-07-17

**Authors:** Yichen Zhu, Chengnan Guo, Hong Fan, Xinyu Han, Yi Li, Xingdong Chen, Tiejun Zhang

**Affiliations:** 1 Shanghai Institute of Infectious Disease and Biosecurity, School of Public Health, Fudan University, Shanghai 200437, China; 21211020033@m.fudan.edu.cn (Y.Z.); cnguo22@m.fudan.edu.cn (C.G.); 20111020010@fudan.edu.cn (H.F.); 21211020076@m.fudan.edu.cn (X.H.); 21211020005@m.fudan.edu.cn (Y.L.); xingdongchen@fudan.edu.cn (X.C.); 2 Key Laboratory of Public Health Safety, Department of Epidemiology, School of Public Health, Ministry of Education, Fudan University, Shanghai 200437, China; 3 Fudan University Taizhou Institute of Health Sciences, Taizhou 176002, China; 4 State Key Laboratory of Genetic Engineering and Collaborative Innovation Center for Genetics and Development, School of Life Sciences, Fudan University, Shanghai 200438, China; 5 Human Phenome Institute, Fudan University, 825 Zhangheng Road, Shanghai 200437, China

**Keywords:** BCAA, cirrhosis complications, prospective cohort study

## Abstract

Background and Aims: The role of serum branched-chain amino acids (BCAAs) in long-term liver cirrhosis complication events remains unclear. We aimed to evaluate the associations between serum BCAAs and the risk of liver-related events. Methods: We included a total of 64,005 participants without liver cirrhosis complication events at baseline from the UK Biobank. Cox proportional hazards regression models were utilized to estimate multivariable hazard ratios (HRs) and 95% CIs for the incidence of liver cirrhosis complication events, adjusting for potential confounders, including sociodemographic and lifestyle factors. Relationships between serum BCAAs and liver cirrhosis complications were examined using nonparametrically restricted cubic spline regression. Results: During a median follow-up of 12.7 years, 583 participants developed liver cirrhosis complication events. The multivariable Cox regression model suggested that total BCAAs (HR  =  0.88, 95% CI 0.82–0.95), serum leucine (HR  =  0.88, 95% CI 0.81–0.95), serum isoleucine (HR  =  0.88, 95% CI 0.82–0.96), and serum valine (HR  =  0.87, 95% CI 0.82–0.96) were all independent protective factors for liver cirrhosis complications after adjustment for sociodemographic and lifestyle factors. Cox models with restricted cubic splines showed U-shaped associations between serum valine and liver cirrhosis complication incidence. Serum total BCAA and isoleucine concentrations might reduce the risk of liver cirrhosis complications by raising the risk of (type 2 diabetes mellitus) T2DM. Conclusion: Lower serum BCAA levels exacerbate the long-term risk of liver cirrhosis complications. Future studies should confirm these findings and identify the biological pathways of these associations.

## 1. Introduction

Liver cirrhosis is the 11th most common cause of death, the third leading cause of death in people aged 45–64 years, and combined with liver cancer, accounts for 3–5% of all deaths worldwide [1,2]. Cirrhosis develops after a long period of inflammation; it evolves from an asymptomatic phase (compensated cirrhosis) to a symptomatic phase (decompensated cirrhosis), the complications of which often result in hospitalization, impaired quality of life, and high mortality [2]. Complications of liver cirrhosis, such as decompensated cirrhosis and primary liver cancer, might lead to subsequent death [3]. 

The liver is a central organ for regulating metabolism; thus, liver cirrhosis frequently results in metabolic alteration. Previous studies have shown that the severity of chronic liver disease could be reflected in a decrease of branched-chain amino acids (BCAAs), which comprise leucine, valine, and isoleucine and together make up about 20% of the total protein content and account for one-third of dietary essential amino acids [4,5,6]. Although previous studies have shown that supplementation with BCAAs may significantly improve prognosis for patients with liver cirrhosis, intervention studies yielded inconsistent results [7,8]. Several randomized controlled trial (RCT) studies conducted in Asian populations showed that oral supplementation with BCAAs could effectively reduce the incidence of serious cirrhotic complications [9,10]. On the basis of a prospective study that enrolled 299 patients from 14 medical institutions in Japan, BCAA supplementation reduced the risk for hepatocellular carcinoma (HCC) and prolonged the survival of patients with cirrhosis [11]. A clinical trial including 39 Japanese patients found that only the subgroup with a baseline serum albumin level of <4.0 g/dL showed lower HCC incidence in BCAA-treated patients [12]. In addition, for cirrhotic patients receiving BCAA supplementation, some studies did not find a significant effect in the occurrence rates of hepatocellular carcinoma [13,14]. The great heterogeneity between previous studies may result from error-prone BCAA supplementation assessments using self-report questionnaires or food records [15]. In contrast, objectively assessed circulating BCAAs are not subject to recall bias and represent the combined effect of dietary intakes and biological processes in vivo [16]. To date, epidemiological studies, especially prospective evidence on serum BCAAs and liver cirrhosis complication incidences, are limited, and no credible evidence has been given.

This study aimed to evaluate whether concentrations of serum BCAAs were associated with the long-term risk of liver cirrhosis complication incidence by leveraging a large population prospective cohort study.

## 2. Materials and Methods

### 2.1. Study Population

We used data from the UK Biobank (application 92718) for a large prospective cohort study of 502,389 participants aged 37 to 73 years recruited from 22 study centers across the United Kingdom (England, Wales, and Scotland) between 2006 and 2010. Participants completed a touchscreen questionnaire, face-to-face interviews, physical measurements, and provided biological samples [17,18]. They gave informed consent to have their records linked to hospital admissions, career registers, and death registers.

For this study, participants at risk for chronic liver disease (CLD) (n = 270,754) were included who met any of the following 4 criteria: (1) alcohol consumption exceeding 14 units/week; (2) abdominal obesity; (3) general obesity; (4) type 2 diabetes mellitus (T2DM). We excluded participants without nuclear magnetic resonance (NMR) metabolomics, as well as those with cirrhosis complication events prior to the interview, leaving 64,005 participants qualified for inclusion in the study. Figure S1 illustrates the flow of patients through the study (Appendix A).

### 2.2. Assessment of Serum BCAAs

Metabolic biomarkers, including BCAAs, were measured from randomly selected EDTA plasma samples (aliquot 3) using a high-throughput NMR-based metabolic biomarker profiling platform developed by Nightingale Health Ltd. (Helsinki, Finland). The measurements took place between June 2019 and April 2020 (Phase 1) and April 2020 and June 2022 (Phase 2) using eight spectrometers at Nightingale Health, based in Finland. The details of these assessments were found on the UK Biobank website (https://biobank.ndph.ox.ac.uk/ukb/label.cgi?id=220, accessed on 20 April 2020).

### 2.3. Ascertainment of Liver Cirrhosis Complications

The primary outcome event was the incidence of a cirrhosis complication event. We defined this as either a hospital admission for cirrhosis, death from cirrhosis, or presentation with HCC [3].

Only events developing during the first 10 years of follow-up were considered. Hospital admissions due to cirrhosis were identified using a validated set of ICD & Operations/Procedure (OPCS4) codes; HCC was defined as the presence of an ICD10:C22.0 code within either a cancer registration or hospital admission record (Table S1) [19]. 

At the time of the present studies, hospital inpatient records were updated until 30 November 2020, and mortality data were available through 18 December 2020 for England and Wales and 10 December 2020 for Scotland. Every participant in the follow-up was censored at the first event of cirrhosis or death, whichever came first. If no events or death occurred, the participants were observed until the end of follow-up.

### 2.4. Assessment of Covariates

A baseline questionnaire was used to assess the following potential confounders: age, gender, ethnicity, Townsend deprivation index (TDI), recruitment center, education, family history of cancer, drinking status, smoking status, physical activity, body mass index (BMI), and T2DM. The TDI was used as an indicator of socioeconomic status, and negative TDI values indicated relative affluence. T2DM was defined as a self-reported history of T2DM, diagnosed T2DM, and diagnosed diabetes, except for T1DM. Participants were classified into low, moderate, and high physical activity degree groups on the basis of categorical criteria in accordance with the guidelines of the International Physical Activity Questionnaire (20). The details of these assessments are available on the UK Biobank website (www.ukbiobank.ac.uk, accessed on 20 April 2020), (Appendix B and Appendix C).

### 2.5. Statistical Analysis

In order to figure out the incidence of liver cirrhosis complication events, person–years were calculated from the date of baseline enrollment until the date of liver cirrhosis diagnosis, date of death, or the end of follow-up, whichever came first. Baseline characteristics were described as the mean and standard deviation (SD) for continuous variables and number (percentage) for categorical variables according to the categories of quartiles of the serum total BCAA concentration. Dose–response relationships were investigated using nonparametrically restricted cubic spline regression with knots at the 25th, 50th, and 75th percentiles between the serum BCAAs concentrations and the risk of liver cirrhosis complications. The serum BCAAs concentrations were then divided into four groups on the basis of quartiles. Each group was modeled as a continuous variable to calculate the *p* for trends. Cox proportional hazards regression was utilized to estimate the hazard ratios and 95% CIs for the prospective associations between serum BCAAs and liver cirrhosis complication incidence. The proportional hazards assumption was tested using Schoenfeld residuals. Baseline age, gender, TDI, ethnicity, recruitment center, education, family history of cancer, smoking status, drinking status, physical activity level, BMI, and T2DM were adjusted. A causal mediation analysis under a counterfactual framework was conducted to calculate the mediation proportion by the mediator (T2DM) for the association between the serum BCAA concentrations and liver cirrhosis complications. Analyses were performed using R.4.3.0. The statistical tests were 2-sided, and *p*-values less than 0.05 were considered statistically significant.

## 3. Results

### 3.1. Baseline Characteristics of the Study Populations

During a median follow-up of 12.7 years, 583 participants had developed liver cirrhosis complications. The total incidence density was 73.83/100,000 person–years. Among people younger than 65 years old, the incidence density was 47.69/100,000 person–years, compared to 76.94/100,000 person–years among people older than 65 years old. The incidence densities of males and females were 89.86/100,000 person–years and 54.60/100,000 person–years, respectively. The overall levels of serum BCAAs in the study populations were 0.356 (95% CI, 0.310 to 0.411) for total BCAA, 0.049 (95% CI, 0.040 to 0.060) for isoleucine, 0.101 (95% CI, 0.087 to 0.119) for leucine, and 0.206 (95% CI, 0.182 to 0.234) for valine. The differences on the concentrations of serum BCAAs between gender groups were statistically significant (*p* < 0.001).

Table 1 shows the baseline characteristics of the participants. Participants with higher levels of total BCAA concentration were more likely to be male and not to report T2DM than those with lower total BCAA concentration. A higher BMI, lower physical activity, and decreasing drinking status were more prevalent among participants with higher levels of total BCAA concentration.

### 3.2. Associations between BCAA Concentration and Liver Cirrhosis Complication Risk

**Figure 1:**
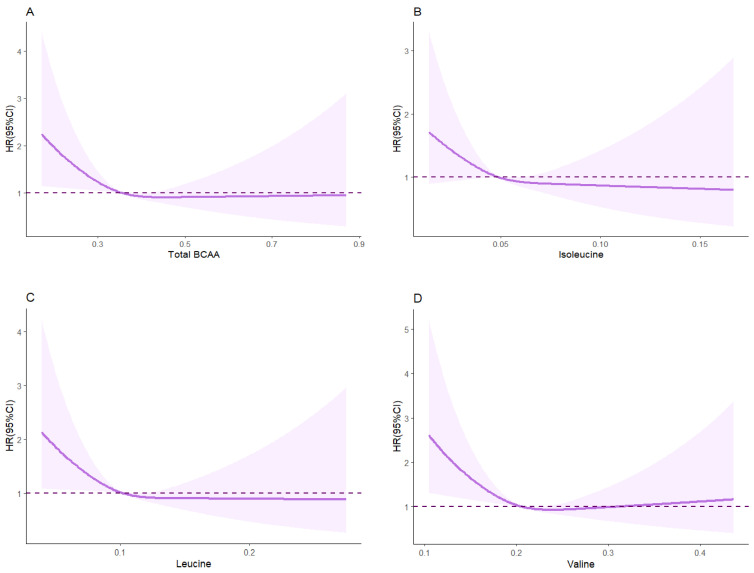
The relationships between BCAA concentrations and liver cirrhosis complications. The results were estimated by restricted cubic spline regressions adjusted for age, gender, BMI, T2DM, ethnicity, Townsend deprivation index, recruitment center, physical activity, drinking status, smoking status, education, and family history of cancer. Shaded areas represent 95% CIs. HR, hazard ratio. (**A**–**D**) represents the relationships between total BCAA, Isoleucine, Leucine and Valine concentrations and liver cirrhosis complications.

We categorized the total BCAA concentration into four groups; compared to the lowest quartile, participants with higher levels of total BCAA concentrations had lower risks of liver cirrhosis complication incidence after adjustment for age, gender, BMI, and T2DM with respective hazard ratios of 0.78 (95% CI, 0.62 to 0.98), 0.67 (95% CI, 0.53 to 0.85), and 0.65 (95% CI, 0.51 to 0.82). After adjustment for age, gender, BMI, T2DM, ethnicity, TDI, recruitment center, physical activity, drinking status, smoking status, education, and family history of cancer, only participants with higher levels of leucine had decreased risks of liver cirrhosis complications, with respective hazard ratios of 0.73 (95% CI, 0.58 to 0.92), 0.70 (95% CI, 0.56 to 0.89), and 0.66 (95% CI, 0.52 to 0.84). The relationships between total BCAA, isoleucine, and valine were less stable and inconsistent, with wide 95% CIs that included 1.0 in some cases (Table 2). Cox models with restricted cubic splines revealed statistically significant U-shaped associations between the serum valine concentration and liver cirrhosis complication incidence after adjusting for age, gender, BMI, T2DM, ethnicity, TDI, recruitment center, physical activity, drinking status, smoking status, education, and family history of cancer (*p* < 0.001) (Figure 1).

### 3.3. Mediation Analysis

T2DM competitively mediated the relationships between lower concentrations of total BCAA, isoleucine, and increased liver cirrhosis complication incidence (mediation proportion, −27.43%, 95% CI, −67.86% to −12%; mediation proportion, −21.22%, 95% CI, −76.94% to −10%) after adjustment for age, gender, BMI, ethnicity, TDI, recruitment center, physical activity, drinking status, smoking status, education, and family history of cancer. The serum total BCAA and isoleucine concentration might attenuate the risk of liver cirrhosis complication by raising the risk of T2DM (Table 3).

### 3.4. Subgroup and Joint Effect Analysis

The subgroup analyses showed that the protective relationships between serum total BCAA and the incidence of liver cirrhosis complication were stronger among individuals who had high ALT and AST levels, with the *p*-interaction of 0.038 and 0.005 (Table 4). Similar inverse relationships between serum total BCAA and the incidence of liver cirrhosis complication were also detected across subgroups of ethnicity and smoking status (*p* for interaction > 0.05).

**Figure 2:**
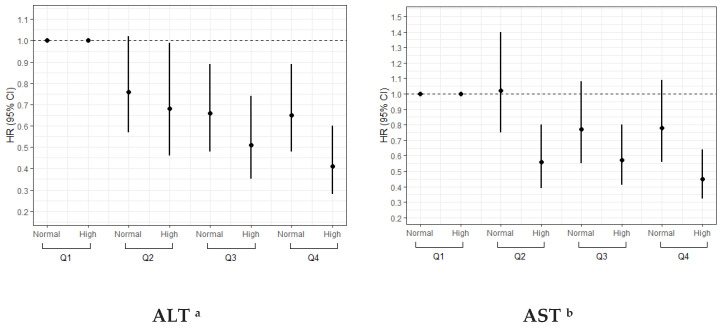
The joint effect of total BCAA with liver cirrhosis complications. ^a^ The results were the joint effect of total BCAA with liver cirrhosis complications for the ALT levels, which is from the Cox proportional hazards model adjusting for age, gender, BMI, T2DM, ethnicity, Townsend deprivation index, recruitment center, physical activity, drinking status, smoking status, education, and family history of cancer. ^b^ The results were the joint effect of total BCAA with liver cirrhosis complications for the AST levels, which is from the Cox proportional hazards model adjusting for age, gender, BMI, T2DM, ethnicity, Townsend deprivation index, recruitment center, physical activity, drinking status, smoking status, education, and family history of cancer.

Meanwhile, similar results were discovered when modeling the serum total BCAA as quartiles. After adjustment for sociodemographic and lifestyle factors, in participants with high ALT and AST levels, the increasing serum total BCAA concentration was more protective compared to the lowest quartile (Figure 2). The interactions between total BCAA and ALT and AST were statistically significant, with *p*-interactions of 0.015 and 0.007.

## 4. Discussion

From this prospective, a population-based cohort of 64,005 participants without prior liver cirrhosis complication events, we found that higher serum BCAA concentrations were associated with a lower risk of liver cirrhosis complication incidence. These associations were independent of measures of potential confounders, including sociodemographic factors and lifestyle behaviors, while the U-shaped curves suggested that the serum valine concentration was a protective factor for cirrhosis complications within a certain range. The results of the subgroup and joint effect analyses revealed that the serum total BCAA was more protective in individuals with higher levels of ALT and AST, suggesting that the supplementation of BCAAs may be helpful to reduce the risk of liver cirrhosis complications in people with higher levels of ALT and AST. The mediation analysis suggested that the serum total BCAA concentration might attenuate the risk of liver cirrhosis complications by increasing the risk of T2DM; thus, patients with T2DM need to be monitored. Few studies have identified the relationships between serum BCAAs and liver cirrhosis complications, and to the best of our knowledge, this is the first cohort study to identify the prospective associations between serum BCAAs and liver cirrhosis complications.

Previous studies have shown that long-term oral supplementation with BCAAs might improve nutritional situations and prevent liver cirrhosis complications [7,8]. There have been no prospective studies that have revealed clear direct relationships between serum BCAAs and liver cirrhosis complication incidences. A recent cohort study revealed the associations between higher serum BCAAs and the occurrence and progression of NAFLD [20]. Another case–control study found that BCAAs were uniformly reduced in plasma [21]. Such a reduction of BCAAs could be explained by the enhanced consumption of BCAA for ammonia detoxication and for energy generation; supplementation with BCAA raises in vitro the synthesis and secretion of albumin by cultured rat hepatocytes without affecting albumin mRNA expression, and BCAAs recover the impaired turnover kinetics of albumin both in the rat cirrhotic model and in cirrhotic patients. Longer-term supplementation with BCAA raises plasma albumin, benefits quality of life issues, and, finally, improves survival in liver cirrhosis [22]. Interestingly, a retrospective study enrolled 158 patients who had been hospitalized with cirrhosis and found that the elevated serum tyrosine concentration, rather than changes in the serum BCAA concentration or the BCAA-to-tyrosine ratio, may indicate a high risk of death or liver transplantation for patients with liver cirrhosis [23], which was limited with its small sample of patients.

BCAAs might decrease the serum ALT levels, as well as alleviate hepatic steatosis and liver injury, by suppressing FAS gene expression, protein levels, and oxidative stress [24,25]. In addition, animal research may explain the biological mechanisms underlying the associations between serum BCAAs and the risk of liver cirrhosis complications. BCAA treatment reduced the translocation of *E. faecalis* through intestinal tight junction recovery and reduced LBP expression in the liver, which repressed the activation of LBP, Toll-like receptor 4, and signal transduction and activator of transcription 3, protecting the liver from cirrhotic injury via multiple pathways [26]. Several events, including an increase in oxidative stress and lipid accumulation, as well as decrease in hepatocyte numbers, are involved in the progression of cirrhosis, while BCAA can attenuate lipotoxic hepatocellular damage in cirrhotic rats through the reduction of lipid peroxides, the recovery of mitochondrial function, and a diminution of hepatocyte death [27]. BCAA supplementation reduced oxidative stress by restoring mitochondrial function and improved iron metabolism by increasing hepcidin-25 in both iron-overloaded HCVTgM and patients with HCV-related advanced fibrosis, which may partially account for their inhibitory effects on HCC development in cirrhosis patients [28]. These biological mechanisms need more validation.

Generally, the serum levels of BCAAs increase in states of obesity and insulin resistance [29,30], and accumulating evidence demonstrates that the presence of T2DM and obesity is associated with an increased risk of hepatic decompensation and HCC [31]. However, we found that the BCAA concentration might attenuate the risk of liver cirrhosis complication by increasing the risk of T2DM. The discrepancy between the results of this study and previous studies might be attributed to the differences in the participant-specific factors, such as the severity of T2DM and level of liver function. Similarly, a RCT conducted in Japan found that a late evening snack (LES) with branched-chain amino acid-enriched nutrients (BCAA-ENs) does not inhibit overt diabetes in most cirrhosis patients, while a BCAA supplementation is effective in improving protein malnutrition in patients with cirrhosis, irrespective of liver disease severity [32]. The reason for this is not fully understood, and further biological mechanistic research is necessary.

This study has several strengths, including its strict quality control with respect to the procedures and data collection, as well as its prospective design, use of objectively measured serum levels of BCAAs, long-time follow up, and large sample size. In addition, this study included only European populations to achieve homogeneity of the study group, which may also be a strength of this research. Our study may have several potential limitations as well. First, given the observational nature of the studies, the possibility of residual confounding such as the dietary- and genetic-related confounders cannot be excluded, which may prevent us from clarifying the causal relationship and mechanisms between BCAAs and cirrhosis complications. Second, the serum BCAAs were evaluated once at baseline, and potential changes over time were not considered.

## 5. Conclusions

In conclusion, our findings demonstrated that serum BCAAs were protective factors for liver cirrhosis complication incidences, particularly for individuals with higher levels of ALT and AST. Our results might provide certain clues for therapeutic treatment; that is, the dietary supplementation of BCAAs is beneficial in the management of cirrhosis complications, with a greater need for those with higher ALT and AST levels. These novel findings are of clinical and public health relevance. A comprehensive understanding of the role of BCAA metabolism in cirrhosis complications, as well as the underlying mechanisms, may pave the way to improving the prevention, diagnosis, and treatment of cirrhosis complications by targeting nutrition and metabolism.

## Figures and Tables

**Table 1:** Baseline characteristics of the participants.

Variables	Overall	Q1 (0.13–0.31)	Q2 (0.31–0.36)	Q3 (0.36–0.41)	Q4 (0.41–1.30)	*p* for Trend ^a^
N	64,005	16,001	16,001	16,002	16,001	
Age (mean (SD))	56.80 (7.94)	56.53 (8.10)	56.96 (7.92)	56.97 (7.89)	56.76 (7.83)	0.015
Gender (%)						<0.001
Female	28,806 (45.0)	10,062 (62.9)	7583 (47.4)	5856 (36.6)	5305 (33.2)	
Male	35,199 (55.0)	5939 (37.1)	8418 (52.6)	10,146 (63.4)	10,696 (66.8)	
Ethnic (%)						<0.001
White	61,480 (96.1)	15,451 (96.6)	15,361 (96.0)	15,373 (96.1)	15,295 (95.6)	
Others	2525 (3.9)	550 (3.4)	640 (4.0)	629 (3.9)	706 (4.4)	
Townsend deprivation index (%)						0.142
1	12,788 (20.0)	3135 (19.6)	3243 (20.3)	3226 (20.2)	3184 (19.9)	
2	12,792 (20.0)	3224 (20.1)	3094 (19.3)	3199 (20.0)	3275 (20.5)	
3	12,826 (20.0)	3154 (19.7)	3221 (20.1)	3271 (20.4)	3180 (19.9)	
4	12,807 (20.0)	3263 (20.4)	3223 (20.1)	3166 (19.8)	3155 (19.7)	
5	12,792 (20.0)	3225 (20.2)	3220 (20.1)	3140 (19.6)	3207 (20.0)	
BMI (mean (SD))	29.48 (5.15)	27.86 (5.20)	29.28 (5.05)	30.04 (5.00)	30.73 (4.92)	<0.001
Drinking status (%)						<0.001
Never	2118 (3.3)	476 (3.0)	509 (3.2)	567 (3.5)	566 (3.5)	
Previous	1765 (2.8)	339 (2.1)	442 (2.8)	424 (2.6)	560 (3.5)	
Current	60,122 (93.9)	15,186 (94.9)	15,050 (94.1)	15,011 (93.8)	14,875 (93.0)	
Smoking status (%)						<0.001
Never	30,140 (47.1)	7529 (47.1)	7640 (47.7)	7413 (46.3)	7558 (47.2)	
Former	26,019 (40.7)	6205 (38.8)	6432 (40.2)	6692 (41.8)	6690 (41.8)	
Current	7846 (12.3)	2267 (14.2)	1929 (12.1)	1897 (11.9)	1753 (11.0)	
Physical activity (%)						<0.001
Low	9242 (14.4)	2120 (13.2)	2241 (14.0)	2373 (14.8)	2508 (15.7)	
Moderate	37,999 (59.4)	9452 (59.1)	9461 (59.1)	9545 (59.6)	9541 (59.6)	
High	16,764 (26.2)	4429 (27.7)	4299 (26.9)	4084 (25.5)	3952 (24.7)	
T2DM (%)						<0.001
Yes	59,614 (93.1)	15,390 (96.2)	15,246 (95.3)	14,836 (92.7)	14,142 (88.4)	
No	4391 (6.9)	611 (3.8)	755 (4.7)	1166 (7.3)	1859 (11.6)	
Education (%)						0.095
No degree	44,468 (69.5)	10,994 (68.7)	11,183 (69.9)	11,130 (69.6)	11,161 (69.8)	
Degree	19,537 (30.5)	5007 (31.3)	4818 (30.1)	4872 (30.4)	4840 (30.2)	
Family history of cancer (%)						0.400
Without	44,384 (69.3)	11,157 (69.7)	11,036 (69.0)	11,133 (69.6)	11,058 (69.1)	
With	19,621 (30.7)	4844 (30.3)	4965 (31.0)	4869 (30.4)	4942 (30.9)	

^a^ The median intake of each quartile was modeled as a continuous variable to calculate the *p*-values for trends.

**Table 2:** Associations between BCAA concentration and liver cirrhosis complication risk.

Variables	Per IQR	Quartile Categories				*p* for Trend ^a^
		Q1	Q2	Q3	Q4	
Total branched-chain amino acids						
Range, mmol/L		0.13–0.31	0.31–0.36	0.36–0.41	0.41–1.30	
Age, gender, BMI and T2DM-adjusted HR ^b^	0.82 (0.75–0.90)	Ref	0.78 (0.62, 0.98)	0.67 (0.53, 0.85)	0.65 (0.51, 0.82)	<0.001
Multivariable-adjusted HR ^c^	0.84 (0.77–0.92)	Ref	0.80 (0.63, 1.00)	0.71 (0.56, 0.90)	0.69 (0.55, 0.88)	<0.001
Isoleucine						
Range, mmol/L		≤0.04	0.04–0.05	0.05–0.06	0.06–0.20	
Age, gender, BMI and T2DM-adjusted HR	0.86 (0.78–0.94)	Ref	0.83 (0.66, 1.04)	0.72 (0.57, 0.92)	0.67 (0.53, 0.86)	<0.001
Multivariable-adjusted HR	0.87 (0.80–0.95)	Ref	0.84 (0.67, 1.05)	0.75 (0.59, 0.95)	0.70 (0.55, 0.89)	0.003
Leucine						
Range, mmol/L		0.03–0.09	0.09–0.10	0.10–0.12	0.12–0.45	
Age, gender, BMI and T2DM-adjusted HR	0.82 (0.75–0.90)	Ref	0.71 (0.56, 0.89)	0.67 (0.53, 0.85)	0.63 (0.50, 0.80)	<0.001
Multivariable-adjusted HR	0.84 (0.77–0.92)	Ref	0.73 (0.58, 0.92)	0.71 (0.56, 0.89)	0.67 (0.52, 0.84)	<0.001
Valine						
Range, mmol/L		0.07–0.18	0.18–0.21	0.21–0.23	0.23–0.85	
Age, gender, BMI and T2DM-adjusted HR	0.82 (0.75–0.90)	Ref	0.87 (0.69, 1.09)	0.68 (0.54, 0.87)	0.69 (0.54, 0.87)	<0.001
Multivariable-adjusted HR	0.84 (0.77–0.92)	Ref	0.90 (0.71, 1.13)	0.72 (0.56, 0.91)	0.73 (0.58, 0.93)	0.003

Abbreviation: HR, hazard ratio; CI, confidence interval; IQR, interquartile range; T2DM, type 2 diabetes mellitus; Ref, reference. ^a^ The median intake of each quartile was modeled as a continuous variable to calculate the *p*-values for trends. ^b^ The results were from the Cox proportional hazards model adjusting for age, gender, BMI, and T2DM. ^c^ The results were from the Cox proportional hazards model additionally adjusting for ethnicity, Townsend deprivation index, recruitment center, physical activity, drinking status, smoking status, education, and family history of cancer.

**Table 3:** Mediation analysis of T2DM on associations of serum BCAAs with liver cirrhosis complication events.

Prop. Mediated (Average)	Estimate	95% CI Lower	95% CI Upper	*p*-Value
Total branched-chain amino acids	−27.43%	−67.86%	−12%	0.04
Isoleucine	−21.22%	−76.94%	−10%	0.02
Leucine	−21.78%	−136%	30%	0.06
Valine	−28.40%	−154%	19%	0.06

**Table 4:** Subgroup analyses and joint effect.

Variables	Count	Percent	*p*-Value	HR ^a^ (95% CI)	*p* for Interaction
Ethnic					0.128
White	61,480	96.1	<0.001	0.83 (0.76, 0.91)	
Others	2525	3.9	0.032	0.49 (0.26, 0.94)	
Townsend deprivation index					0.007
1	12,788	20	0.541	0.93 (0.73, 1.18)	
2	12,792	20	0.852	0.98 (0.80, 1.21)	
3	12,826	20	0.126	0.85 (0.68, 1.05)	
4	12,807	20	0.023	0.78 (0.63, 0.97)	
5	12,792	20	<0.001	0.74 (0.63, 0.86)	
Ever drinking					0.307
No	2118	3.3	0.468	1.19 (0.74, 1.91)	
Yes	61,887	96.7	<0.001	0.81 (0.74, 0.89)	
Ever smoking					0.479
No	30,140	47.1	0.013	0.82 (0.70, 0.96)	
Yes	33,865	52.9	<0.001	0.82 (0.74, 0.92)	
Physical activity					0.020
Low	9242	14.4	<0.001	0.68 (0.55, 0.83)	
Moderate	37,999	59.4	0.003	0.83 (0.74, 0.93)	
High	16,764	26.2	0.323	0.91 (0.76, 1.10)	
Education					0.215
No degree	44,468	69.5	<0.001	0.81 (0.73, 0.90)	
Degree	19,537	30.5	0.107	0.85 (0.71, 1.04)	
Family history of cancer					0.127
Without	44,384	69.3	<0.001	0.78 (0.69, 0.87)	
With	19,621	30.7	0.199	0.91 (0.78, 1.05)	
ALT					0.045
Normal	56,711	88.6	<0.001	0.80 (0.71, 0.90)	
High	7294	11.4	<0.001	0.70 (0.61, 0.81)	
AST					0.009
Normal	59,764	93.4	0.009	0.85 (0.75, 0.96)	
High	4241	6.6	<0.001	0.73 (0.64, 0.83)	

Abbreviations: HR, hazard ratio; CI, confidence interval; ALT, alanine aminotransferase; AST, aspartate transaminase. ^a^ The results were from the Cox proportional hazards model adjusted for age, gender, BMI, and T2DM.

## Data Availability

The data which support the findings of this study are available in UK Biobank—https://www.ukbiobank.ac.uk/ (accessed on 20 April 2020).

## Supplementary Materials

The following supporting information can be downloaded at https://www.mdpi.com/article/10.3390/nu16142295/s1. Figure S1. The flowchart of the study design and participants excluded from the study. Table S1. ICD 10 and OPCS4 codes used to define cirrhosis-related complication events.

## Abbreviations

Abbreviation	Definition
BCAA	branched-chain amino acids
T2DM	type 2 diabetes mellitus
CLD	chronic liver disease
TDI	townsend deprivation index
BMI	body mass index
HR	hazard ratio
CI	confidence interval
IQR	interquartile range
ALT	alanine aminotransferase
AST	aspartate transaminase

## Appendix A. Variables Used to Define the Study Population

Each participant’s BMI was calculated based on their weight and height data. The Tanita BC-418 MA body composition analysis was used to determine body weight, and the Seca202 height measure was used to measure standing height [33].

During the interview of UK biobank, a computer-assisted touchscreen interface was utilized to collect data on alcohol intake. Participants were requested to provide information about their weekly or monthly average alcohol intake, including the number of glasses of red wine (Field IDs: 1568, 4407), glasses of champagne/white wine (Field IDs: 1578, 4418), pints of beer/cider (Field IDs: 1588, 4429), measures of spirits (Field IDs: 1598, 4440), glasses of fortified wine (Field IDs: 1608, 4451), and glasses of other types of alcoholic drinks (Field IDs: 5364, 4462). In order to produce more accurate estimates for infrequent drinkers, the consumption of alcohol was recorded as an “average month” for those who did not consume alcohol on a weekly basis or who just occasionally consumed. To determine the average number of alcohol units consumed by each participant each week, we estimated that there are 2 units (16 g) of pure alcohol in a pint of beer/cider; 1.5 units (12 g) in a glass of red wine, champagne, white wine, fortified wine, and “other” alcoholic drink; and 1 unit (8 g) in a measure of spirits. These conversion values are consistent with the methods protocol used in the Health Survey for England [34].

When UK biobank participants were inquired about their diagnosis of diabetes mellitus (Field ID: 2443), they were not specifically asked about type 2 diabetes (T2DM). Consequently, we inferred the T2DM status by taking into account all individuals who have reported a diabetes diagnosis (Field ID: 2443) and excluding those with evidence of non-type 2 diabetes. Evidence of non-type 2 diabetes was based on either: (1) self-reported type 1 diabetes in the UK biobank nurse interview, (2) hospital admission for type 1 diabetes (ICD10: E10), OR (3) self-reported gestational diabetes (field ID: 4041).

## Appendix B. The Lists of Demographic Factors and Laboratory Metrics

Basic demographic information, including age, gender, Townsend deprivation index (TDI), ethnicity, waist circumference, hip circumference, waist/hip ratio, height, weight, BMI, pulse rate, diastolic blood pressure, systolic blood pressure, body fat percentage, basal metabolic rate, pre-existing medical conditions (T2DM, hypertension, and cardiovascular disease), vitamin and mineral supplement use; lifestyle information (alcohol drinker status, alcohol consumption per week, smoking status, sleep duration, and physical activity over the past week), was extracted from the questionnaire, interview at recruitment, primary care records and hospital episode statistics. The TDI was an indication of socioeconomic status, and negative values denoted relative affluence. Physical activity was divided into three levels based on categorical criteria, following the guidelines of the International Physical Activity Questionnaire (Appendix C) [35].

Laboratory metrics with evidence of prognostic ability for liver cirrhosis complications were enrolled in the current study (with more than 5 different risk scores cited), including albumin, alanine aminotransferase (ALT), AST, total bilirubin, and PLT [3]. These laboratory metrics were measured by BCG analysis on a Beckman Coulter AU5800 using blood specimens collected at baseline interview.

## Appendix C. Categorical Criteria of Physical Activity Level

Category 1 Low

Those participants who have not met criteria for Categories 2 or 3 are considered to have a ‘low’ physical activity level.

Category 2 Moderate

Either of the following 3 criteria:

(1) 3 or more days of vigorous-intensity activity of at least 20 min per day;
(2) 5 or more days of moderate-intensity activity and/or walking of at least 30 min per day;
(3) 5 or more days of any combination of walking, moderate-intensity or vigorous intensity activities achieving a minimum Total physical activity of at least 600 MET-minutes/week.

Category 3 High

Any one of the following 2 criteria:

(1) vigorous-intensity activity on at least 3 days achieving a minimum Total physical activity of at least 1500 MET-minutes/week;
(2) 7 or more days of any combination of walking, moderate-intensity or vigorous-intensity activities achieving a minimum Total physical activity of at least 3000 MET-minutes/week.
