# Supplementary material for: Serum Branched-Chain Amino Acids and Long-Term Complications of Liver Cirrhosis: Evidence from a Population-Based Prospective Study
Source: Nutrients. 2024 Jul 17;16(14):2295. doi: 10.3390/nu16142295 (PMC11279618; doi:10.3390/nu16142295)
Supplement: Supplementary file 1 [file nutrients-16-02295-s001.zip › nutrients-3087424-supplementary.pdf]

Original Article

Serum branched-chain amino acids and long-term complications of liver cirrhosis: evidence from the population-based UK Biobank study

Yichen Zhu[1,2†], Chengnan Guo[1†], Tiejun Zhang[1,2]

1. Department of Epidemiology, School of Public Health, Fudan University, Shanghai, China.

2. Fudan University Taizhou Institute of Health Sciences, Taizhou, China.

Running title: Serum BCAAs and liver cirrhosis complication

† These authors contributed equally to this work.

* Correspondence to Tiejun Zhang MD., PhD., Professor of Epidemiology, School of Public Health, Fudan University, Shanghai, 200032, China. Email: tjzhang@shmu.edu.cn ; TEL/FAX: +86−21−54237088;

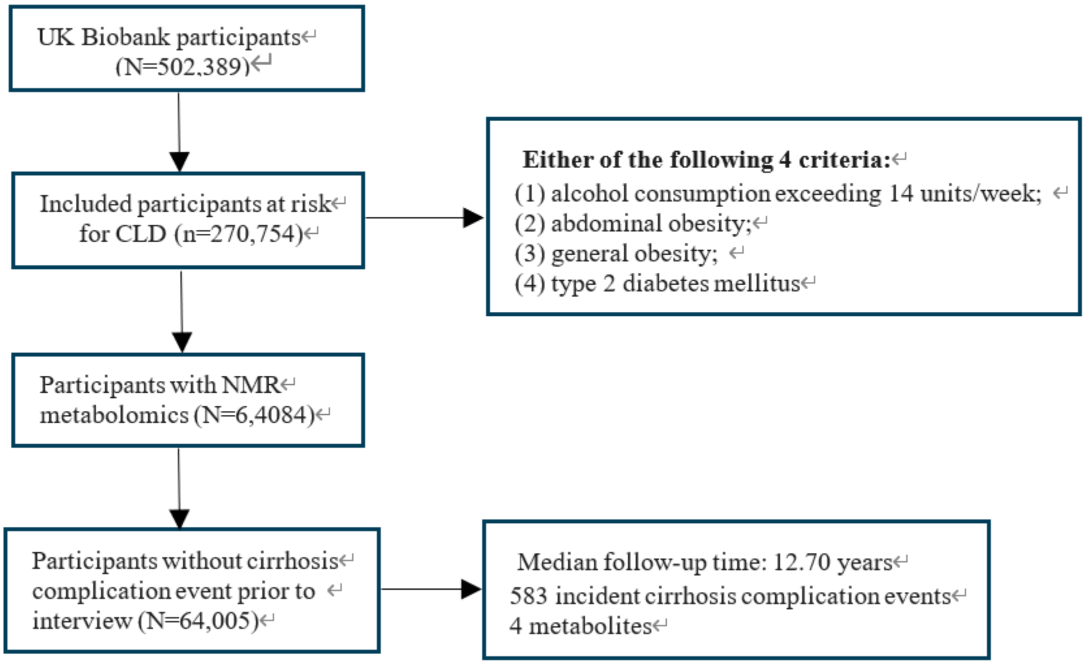

Figure S1. The flowchart of the study design and participants excluded from the study

Table S1. ICD 10 and OPCS4 codes used to define cirrhosis-related complication events

Type of complication event	Code type	Code	Code description
Cirrhosis-related hospital admission	ICD 10	K70.3	Alcoholic cirrhosis of liver
		K71.7	Toxic liver disease with fibrosis and cirrhosis of liver
		K72.1	Chronic hepatic failure
		K74.4	Secondary biliary cirrhosis
		K74.5	Biliary cirrhosis, unspecified
		K74.6	Other and unspecified cirrhosis of liver
		K76.6	Portal hypertension
		I85.0; I859; I98.2; I98.3	Esophageal varices
		I86.4	Gastric varices
	OPCS4	J06.1	Tranjugular intrahepatic insertion of stent into portal vein
		J06.2	Transjugular intrahepatic insertion of stent graft into portal vein
		G10.4	Local ligation of varices of oesophagus
		G10.8	Other specified open operations on varices of oesophagus
		G10.9	Unspecified open operations on varices of oesophagus
		G14.4	Fibreoptic endoscopic injection sclerotherapy to varices of oesophagus
		G17.4	Endoscopic injection sclerotherapy to varices of oesophagus using rigid oesophagoscope
		G43.7	Fibreoptic endoscopic rubber band ligation of upper gastrointestinal tract varices
		T46.1*	Paracentesis abdominis for ascites
		T46.2*	Drainage of ascites not elsewhere specified
Hepatocellular carcinoma	ICD 10	C22.0	Liver cell carcinoma
Cirrhosis-related death	ICD 10	K70.3	Alcoholic cirrhosis of liver
		K71.7	Toxic liver disease with fibrosis and cirrhosis of liver
		K72.1	Chronic hepatic failure
		K74.4	Secondary biliary cirrhosis
		K74.5	Biliary cirrhosis, unspecified
		K74.6	Other and unspecified cirrhosis of liver
		K76.6	Portal hypertension
		I85.0; I859; I98.2	Esophageal varices
		I86.4	Gastric varices
		C22.0	Liver cell carcinoma

ICD-10 refers to International Classification of Disease version 10. OPCS4 refers to Operation/procedure codes version 4. A complication event was considered to be due to cirrhosis morbidity if any of the above codes were present in any diagnostic or cause of death position. However, the OPCS4:T461 and OPCS4:T462 (codes relating to ascites) codes are exceptions to this rule. Here, these codes were only considered to reflect cirrhosis morbidity if accompanied by at least one corroborating ICD code for chronic liver disease (i.e., ICD10: K70-K77). This qualification is necessary because ascites can have non-hepatic causes.
